# Information from ecological momentary assessments lead to over-medicalization: Commentary

**DOI:** 10.1177/13524585241253081

**Published:** 2024-05-15

**Authors:** Daan J de Jong, Eva M Strijbis, Joep Killestein

**Affiliations:** MS Center Amsterdam, Department of Neurology, Vrije Universiteit Amsterdam, Amsterdam Neuroscience, Amsterdam UMC location VUmc, Amsterdam, The Netherlands MS Center Amsterdam, Department of Anatomy and Neurosciences, Vrije Universiteit Amsterdam, Amsterdam Neuroscience, Amsterdam UMC location VUmc, Amsterdam, The Netherlands; MS Center Amsterdam, Department of Neurology, Vrije Universiteit Amsterdam, Amsterdam Neuroscience, Amsterdam UMC location VUmc, Amsterdam, The Netherlands; MS Center Amsterdam, Department of Neurology, Vrije Universiteit Amsterdam, Amsterdam Neuroscience, Amsterdam UMC location VUmc, Amsterdam, The Netherlands

Conventional care diagnostics might be insufficient in fully capturing the variety of symptoms in people with multiple sclerosis (PwMS), as they fluctuate over time and conventional outcome measures are prone to bias.^1–3^ Hence, there is increasing focus on developing methods that collect ecological momentary assessments (EMAs). EMA is a broad collection of applications measuring real-time and real-world data.^3^ Examples range from digital biomarkers that are passively acquired by the patient’s mobile device such as keystroke dynamics,^4^ to methods that require active interaction such as tests for cognition and ambulation.^5–7^ With the emergence of these new diagnostics, concerns arise about the effects on health care and well-being of PwMS. In this edition of controversies, Preziosa et al.^8^ and Høgestøl and Berg-Hansen^9^ contribute with opposing statements on the effect of EMA on over-medicalization.

The authors build their arguments on a shared foundation; both statements argument that EMA enhance the understanding of the fluctuation of factors that influence multiple sclerosis (MS) symptoms, and the way symptoms impact performance, ultimately, contributing to a better understanding of pathophysiological substrates in MS and delivering personalized care. However, the authors identify different outcomes.

Høgestøl and Berg-Hansen^9^ raise concerns about the quality of the newly acquired data that necessitates physicians to careful interpretation. Health care providers need to be aware of the pitfalls of real-world data that are susceptible to bias and sources of noise that may not have been considered in clinical consultation before. An example is the improved performance in electronic cognition tests after repeated use, caused by a practice effect.^5^ Høgestøl worries misinterpreting the results might lead to over-medicalization. However, an advantage of EMA may lie within this drawback. Preziosa suggests the increased frequency of testing can positively impact the signal-to-noise ratio. Frequent measures that reduce this ratio and de-noising methods that require the frequent data,^5^ aid the timely detection of subtle change and facilitate the timely implementation of preventive pharmaceutical or non-pharmaceutical interventions. They hypothesize this could tailor future need for symptomatic treatment. After all, the increase in symptoms would also have taken place in the absence of EMA, early detection makes it possible to act earlier using alternative approaches.

Intriguingly, both statements shed light on an underlying discussion surrounding the role of PwMS in their own care: a discussion on the value of freedom in a field that increasingly requests for autonomy.^10^ The multifaceted nature of this discussion is underscored by the authors. Høgestøl is concerned that the use of EMA itself causes over-medicalization, as constant monitoring might contribute to stress and be perceived as a time burden. While opposing views suggest that EMA might also empower PwMS to increase self-efficacy.^10^

Importantly, the role and the applicability of EMA in MS is still a developing field. To prevent over-medicalization, future advances should provide guidance in this discussion, underpinning the wishes of both physicians and patients, and warranting the strengths and pitfalls of the novel monitoring methods. Notable studies that aim to provide insights in this discussion are the second phase of the DreaMS validation study^7^ and the continuation of the APPSMS study (the Connect-MS).^4,5^
